# A hybrid blockchain migration framework for converting traditional databases into blockchain-based EMR systems

**DOI:** 10.1038/s41598-026-36787-6

**Published:** 2026-02-05

**Authors:** Ahmed Al-Busaidi, Joseph Mani, Mohamed Sirajudeen Yoosuf, Vijaya P

**Affiliations:** Department of Mathematics and Computer Science, Modern College of Business and Science, Muscat, Oman

**Keywords:** Electronic medical records, Blockchain, Hybrid database, Hyperledger fabric, OpenMRS, Data migration, Healthcare IT, Data integrity, Oman PDPL, Compliance, Computational biology and bioinformatics, Engineering, Health care, Mathematics and computing

## Abstract

Electronic Medical Records (EMRs) are crucial to modern healthcare. However, traditional relational databases fail to fulfill increased expectations for integrity, auditability, and compliance in regulated environments. This paper proposes a Hybrid Blockchain Migration Framework that integrates a conventional MySQL-based EMR system (OpenMRS) with a permissioned blockchain network (Hyperledger Fabric). Sensitive data fields are selectively mirrored to the blockchain, ensuring tamper-evident logging while retaining the high performance of SQL for routine operations. A middleware layer, implemented using Java Spring Boot, monitors changes in the EMR and commits cryptographic hashes and metadata to the blockchain in near real-time. We evaluate the hybrid system against both standalone MySQL and full-blockchain implementations using controlled benchmarks, analyzing latency, throughput, resource utilization, and auditability. Results show that the hybrid architecture sustains near-native responsiveness (median 2.1 ms versus 1.6 ms for pure MySQL and 60.5 ms for Fabric) and delivers 480 Transaction Per Second (TPS), while incurring only modest overhead (47% of i7-9750H CPU, 1.15 GB RAM) and enhancing data integrity and compliance with regulations such as Oman’s Personal Data Protection Law (PDPL). The framework is extensible to multi-institutional deployments and supports regulatory alignment, making it a viable pathway for blockchain adoption in clinical settings.

## Introduction

Healthcare systems worldwide are undergoing a rapid digital transformation, with Electronic Medical Records (EMRs) becoming the primary reference for each encounter in a patient’s lifelong care journey. Modern EMRs must therefore satisfy two seemingly conflicting requirements: (i) deliver millisecond-level response times for clinicians, and (ii) guarantee uncompromised integrity, auditability, and seamless data exchange across institutions.

**Figure 1:**
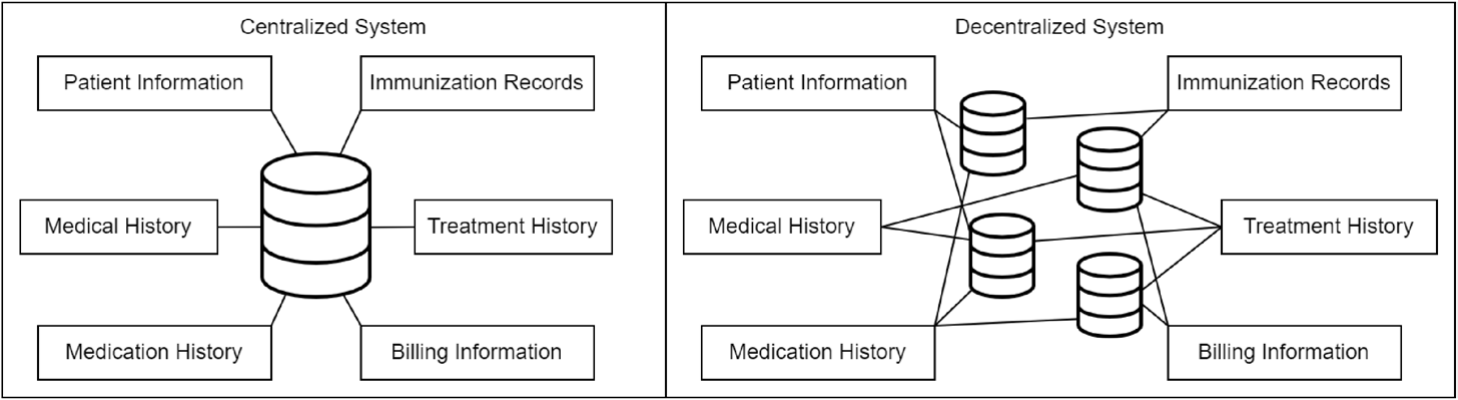
Visual comparison of a single-point, centralised EMR database (left) with the proposed decentralised / hybrid approach (right). The decentralised model distributes data storage across blockchain peers, mitigating a single point of failure while improving auditability.

The majority of existing deployments still rely on a *single, centralised relational database* (left), as seen in Fig. 1. This architecture creates a critical single point of technological and governance failure, as all operational modules, including registration, billing, laboratory, and pharmacy, write to the same core system. In such environments, years of clinical history can be silently corrupted or rendered inaccessible during large-scale incidents such as the WannaCry ransomware attack, which disrupted hospital systems worldwide, or through unexpected EMR platform outages reported in major vendors ^19,20^. Even minor configuration errors introduced by privileged administrators can cascade into system-wide data loss. Because routine backups often replicate the compromised state, organizations may be unable to trace the root cause or recover the original records.

The *hybrid architecture* suggested in this work is sketched on the right side of Fig. 1. While some data, such as patient identification, medications, and diagnoses, are mirrored to a permissioned Hyperledger Fabric ledger, routine CRUD operations continue to take place on a high-performance MySQL. This division adds a tamper-evident record-keeping layer that auditors, regulators, and even patients may independently verify while maintaining the responsiveness that doctors require.

Thus, rather than replacing blockchain technology entirely, it acts as a supplementary *trust anchor*. Its fundamental characteristics (traceability, immutability, and decentralization) align well with the evolving requirements of medical data governance. According to surveys and systematic evaluations^1–3^, blockchain has potential for enabling safe data exchange, removing obstacles to inter-institutional trust, and producing tamper-evident records. However, considering blockchain as a universal solution to all challenges is technically misguided, since moving an entire EMR workload on-chain is impractical due to data volume, privacy regulation, and real-time usability constraints. Therefore, the research community has called for practical *hybrid* solutions that integrate the benefits of both architectures^5,16^.

**Our Contribution.** We propose a **Hybrid Blockchain Migration Framework** that integrates Hyperledger Fabric into an existing MySQL (e.g. OpenMRS) system via a Java Spring Boot middleware. The middleware captures database triggers, hashes critical records, and commits them as immutable transactions, while non-essential fields remain off-chain. The framework includes a GUI-based migration tool, automated trigger and chaincode generators, and a real-time validator to synchronize blockchain and database states. It supports compliance with Oman’s PDPL through verifiable consent logs, fine-grained access auditing, and regulator-facing read-only peers, without disrupting clinical workflows.

**Key Contributions:**

- A modular hybrid migration framework that integrates MySQL-based EMRs with Hyperledger Fabric.
- Selective on-chain storage of data to enable tamper-evident audit trails and data integrity verification.
- Graphical tools for both migration and verification, including auto-generated database triggers and chaincode for streamlined deployment and consistency checks.
- Evaluation through controlled benchmarking and regulatory compliance mapping aligned with Oman’s PDPL.

The remainder of this paper is organized as follows. First section formulates the research problem and outlines key objectives. Section “Related work” reviews relevant literature and identifies research gaps. Section “Proposed framework” presents the hybrid architecture and system design. Section “Implementation details” discusses the technical implementation, including database triggers, chaincode logic, and the middleware pipeline. Section “Compliance with Oman’s healthcare laws” examines legal compliance with healthcare data protection standards. Section “Results and discussion” presents comparative performance results. Section “Conclusion and future work” concludes the paper and outlines future work directions, such as public blockchain anchoring, privacy-preserving extensions, and national-scale deployments.

### Problem statement

Healthcare providers in Oman and worldwide increasingly rely on EMR systems such as OpenMRS, which use relational databases (e.g., MySQL) as their primary data store. OpenMRS alone is deployed in over 8,100 healthcare sites across more than 80 countries^17^, indicating the scale at which such systems underpin clinical operations. While these platforms offer high performance, familiarity, and mature tooling, they exhibit several critical limitations:

- **Auditability**: Logs are centrally stored and not independently verifiable. This introduces a single point of trust and complicates forensic investigations, regulatory audits, and inter-institutional data exchange.
- **Data Integrity**: Administrators or attackers with elevated privileges can alter or delete records without leaving tamper-evident traces. Traditional logging and replication strategies lack immutability guarantees.
- **Regulatory Compliance**: Under Oman’s PDPL^12^, healthcare data is subject to strict consent management, access control, retention, and breach traceability requirements, which are not natively enforced by MySQL-based EMRs.
- **Interoperability**: Siloed, institution-specific databases limit seamless EMR exchange between departments and hospitals, creating operational friction and reducing trust in shared clinical data.

In essence, while current EMR systems perform well operationally, they lack the tamper-evidence, decentralized trust, and compliance alignment needed for modern healthcare ecosystems^9^. Fully migrating to blockchain-based EMRs promises stronger guarantees but introduces latency, architectural complexity, and usability challenges in clinical settings^5^. The core challenge, therefore, is to integrate blockchain with existing EMR platforms in a manner that strengthens security and compliance *without disrupting clinical workflows*.

**Use Case Scenario** A regional hospital in Oman running OpenMRS must ensure that once a doctor records a diagnosis, it cannot be altered without detection, and all access is logged for compliance. Multiple departments interact with shared patient records, and regulators require transparent verification of access and changes. Any solution must support these needs without degrading EMR responsiveness or requiring a complete system overhaul.

### Objectives

This research aims to design a hybrid architecture that combines the scalability of traditional databases with the integrity and auditability of blockchain for managing Electronic Medical Records (EMRs). The specific objectives are:

- **Selective Blockchain Integration**: Mirror critical EMR fields from MySQL onto Hyperledger Fabric to create tamper-evident logs and enhance trust.
- **Preserve Performance and Scalability**: Offload routine data operations to MySQL while limiting blockchain to high-value transactions, ensuring system responsiveness and scalability.
- **Develop a Reusable Migration Toolkit**: Provide a modular framework supporting selective column migration, trigger automation, and chaincode generation, which generalizes to other EMR systems like OpenMRS.
- **Enable Regulatory Compliance**: Implement fine-grained access control, consent tracking, and immutable logs in alignment with Oman’s PDPL and healthcare audit requirements.
- **Benchmark Hybrid vs. Traditional and Full Blockchain Models**: Evaluate integrity, performance, and compliance trade-offs across the three architectures to validate the hybrid approach.

This framework is designed for incremental adoption, enabling healthcare providers to integrate blockchain gradually without overhauling existing systems, a practical path for real-world deployment.

## Related work

Blockchain technology has gained increasing attention in healthcare for its potential to ensure data integrity, transparency, and decentralized trust. Mettler^14^ first introduced the concept of blockchain as a transformative tool for health data management. A notable early implementation is MedRec by Azaria^8^, which used Ethereum to manage access permissions and log events via smart contracts. MedRec introduced the hybrid model of recording access metadata on-chain while keeping sensitive data off-chain, a design pattern that has been widely adopted in later systems.

Daraghmi et al.’s MedChain^4^ similarly emphasized off-chain storage, leveraging encryption and short-term smart contracts to manage data access. While these systems improve privacy and control, they do not address integration with legacy EMR systems like OpenMRS. Our work targets this critical gap.

Blockchain has also been applied to data integrity assurance in specific domains. Ichikawa et al.^6^ demonstrated tamper-resistant logs for mobile health data, while Rouhani et al. proposed MediChain^7^, combining decentralized ledgers with off-chain cloud storage. These efforts reinforce a common consensus: storing only minimal metadata on-chain is essential for scalability^5^.

Several comprehensive reviews^1–3^ have synthesized blockchain applications in healthcare, highlighting benefits such as data immutability, interoperability, and patient empowerment. At the same time, they note significant challenges: limited throughput, privacy concerns, and regulatory compliance requirements. While these studies generally advocate permissioned blockchains such as Hyperledger Fabric for medical contexts, due to fine-grained access control and configurable governance, real-world deployments often reveal that fully permissionless or wholly on-chain solutions struggle to meet performance and compliance demands. A prominent real-world example is Estonia’s nationwide e-Health system: the e-Estonia program employs KSI Blockchain to cryptographically ensure the integrity and auditability of patient records, while the primary record storage continues to reside in traditional relational databases (e.g., Oracle)^18^. This hybrid model illustrates the practical value and feasibility of selective blockchain adoption in healthcare, guiding our own design choice.

Closer to our focus, Guo et al.^10^ proposed a hybrid blockchain-edge model for electronic health record (EHR) access control, using edge servers for computation and blockchain for audit trails. Wang et al.^11^ developed a Fabric-based framework for interoperable healthcare data sharing, though their approach assumes a fresh deployment without existing infrastructure. In contrast, our approach supports incremental integration with legacy SQL-based EMRs, lowering adoption barriers. Privacy-preserving blockchain frameworks have also emerged: Stamatellis et al.^13^ employed Identity Mixer (Idemix) on Hyperledger Fabric for anonymous credential management, while Lee et al.^15^ emphasized consent-driven patient data exchange. These studies guide our roadmap for future enhancements, although our current system limits blockchain exposure to hashed and minimal data within a closed, permissioned network.

In summary, the literature indicates that:

1. Blockchain improves EMR data integrity, traceability, and access control.
2. Fully on-chain architectures are generally impractical at scale.
3. Hybrid approaches (combining blockchain with existing infrastructures) are widely recommended for real-world deployment^5,7^.

To our knowledge, no prior work offers a comprehensive blueprint for integrating OpenMRS with a blockchain backend. This paper addresses that gap by presenting a modular, tested framework that augments legacy EMRs with blockchain functionality, without requiring full system replacement. Table 1 compares our approach with recent blockchain-based healthcare data-management frameworks cited in this paper, highlighting the absence of migration support and system-level integration in prior work.

**Table 1:** Comparison of recent blockchain-based healthcare data-management frameworks.

Work	Data model	Migration support	Compliance target	Year
MedRec ^8^	Off-chain hashes	–	HIPAA	2016
MedChain ^4^	Encrypted off-chain	–	GDPR	2019
MediChain ^7^	Cloud + ledger	–	–	2018
Guo ^10^	Edge + ledger	Partial	–	2019
Wang ^11^	Fabric-based	–	–	2021
Estonia e-Health ^18^	Oracle DB + KSI integrity chain	–	GDPR	2012
**Ours**	Hybrid SQL/ledger	$\checkmark$	PDPL	2025

Only works cited elsewhere in this paper are included.

## Proposed framework

To address the limitations identified in existing EMR systems and their integration with blockchain technologies, we propose a modular **Hybrid Blockchain Migration Framework** that enhances a traditional OpenMRS deployment with permissioned blockchain capabilities. The architecture strengthens data integrity, enables tamper-evident logging, and supports regulatory compliance, particularly with Oman’s Personal Data Protection Law (PDPL), without disrupting clinical workflows.

At a high level, the system consists of three tightly integrated components: (1) the **Data Layer** built on OpenMRS and MySQL, (2) the **Blockchain Layer** powered by Hyperledger Fabric, and (3) a **Middleware Layer** implemented in Java Spring Boot that orchestrates real-time synchronization between the two. Clinicians interact with OpenMRS as usual, while backend processes are augmented with blockchain transparency and auditability.

**Figure 2:**
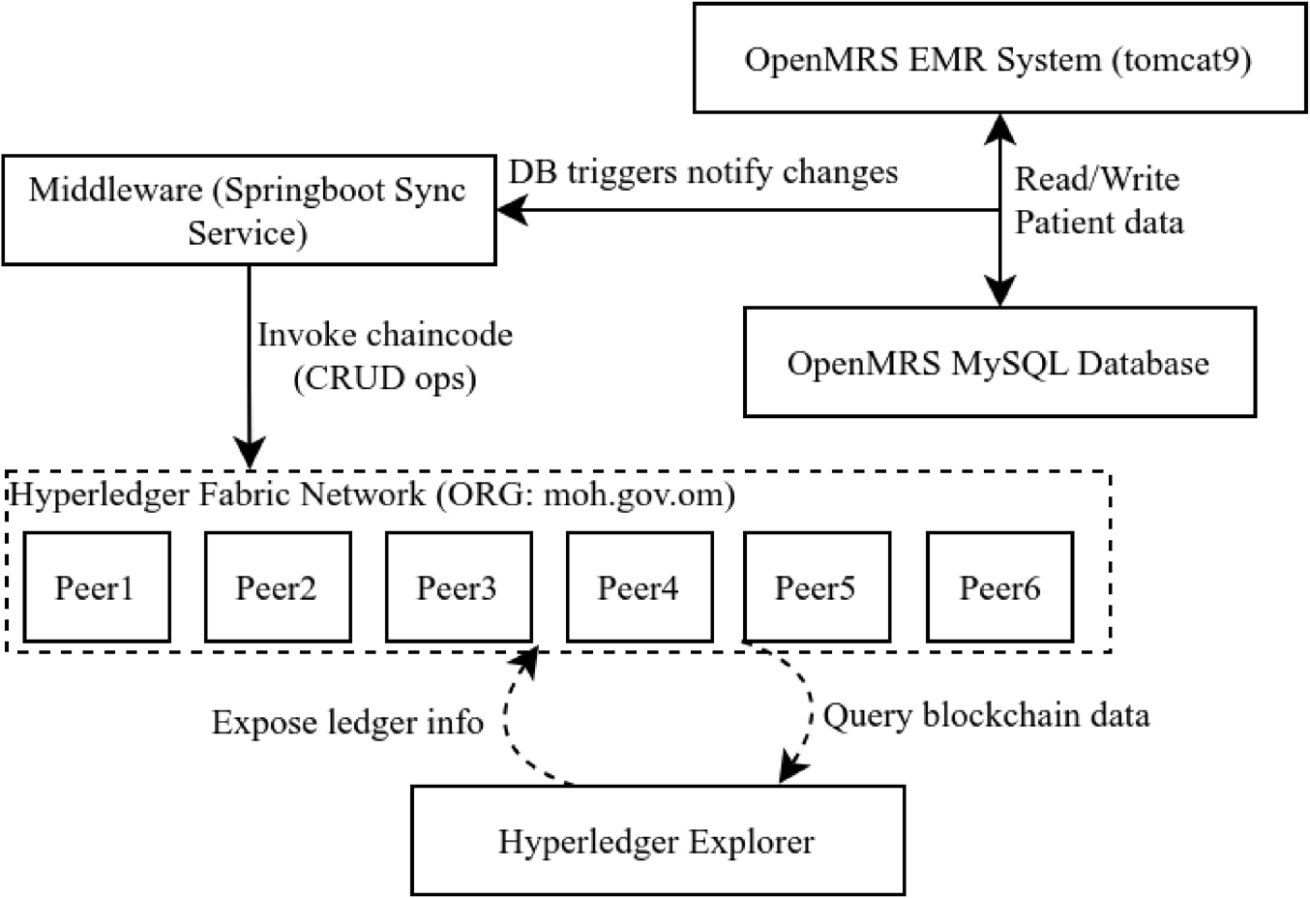
Proposed hybrid blockchain migration framework architecture.

The complete architecture is illustrated in Fig. 2.

- **Data Layer:** Retains the legacy OpenMRS-based EMR system, backed by a MySQL database. This layer handles routine healthcare operations and user interactions, ensuring continuity in clinical workflows.
- **Blockchain Layer:** Implements a permissioned ledger using Hyperledger Fabric. Instead of storing full patient records, it stores cryptographic hashes and transaction metadata to ensure immutability, traceability, and decentralized verification of sensitive operations.
- **Middleware Layer:** Acts as the interface between the EMR database and the blockchain. It monitors SQL triggers for insert and update operations, computes SHA-256 hashes, and invokes relevant chaincode functions. It also manages peer communications, error handling, and synchronization consistency.

**Figure 3:**
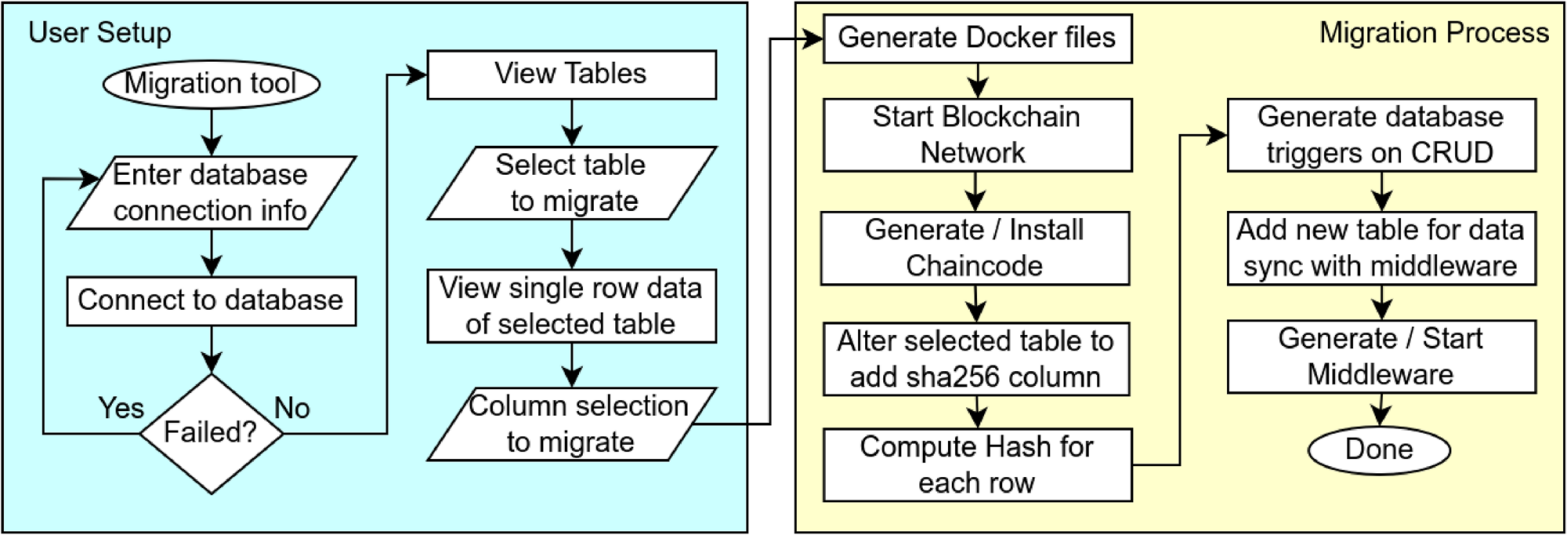
End-to-end migration workflow by the migration tool: user setup (blue) defines connections and table/column selections; migration process (yellow) auto-generates Docker files, deploys Fabric, installs chaincode, adds triggers, and starts middleware.

To streamline system configuration and deployment, a GUI-based migration tool has been developed. It abstracts the underlying blockchain complexities and automates key steps such as network provisioning, smart contract deployment, and trigger generation. Figure 3 illustrates the end-to-end migration workflow.

The **User Setup Lane** (blue) enables configuration of MySQL connection parameters and selection of relevant EMR tables and fields. The **Migration Process Lane** (yellow) automates the generation of Docker and Fabric artifacts, instantiates chaincode, configures SQL triggers, and launches the middleware for synchronization.

**Figure 4:**
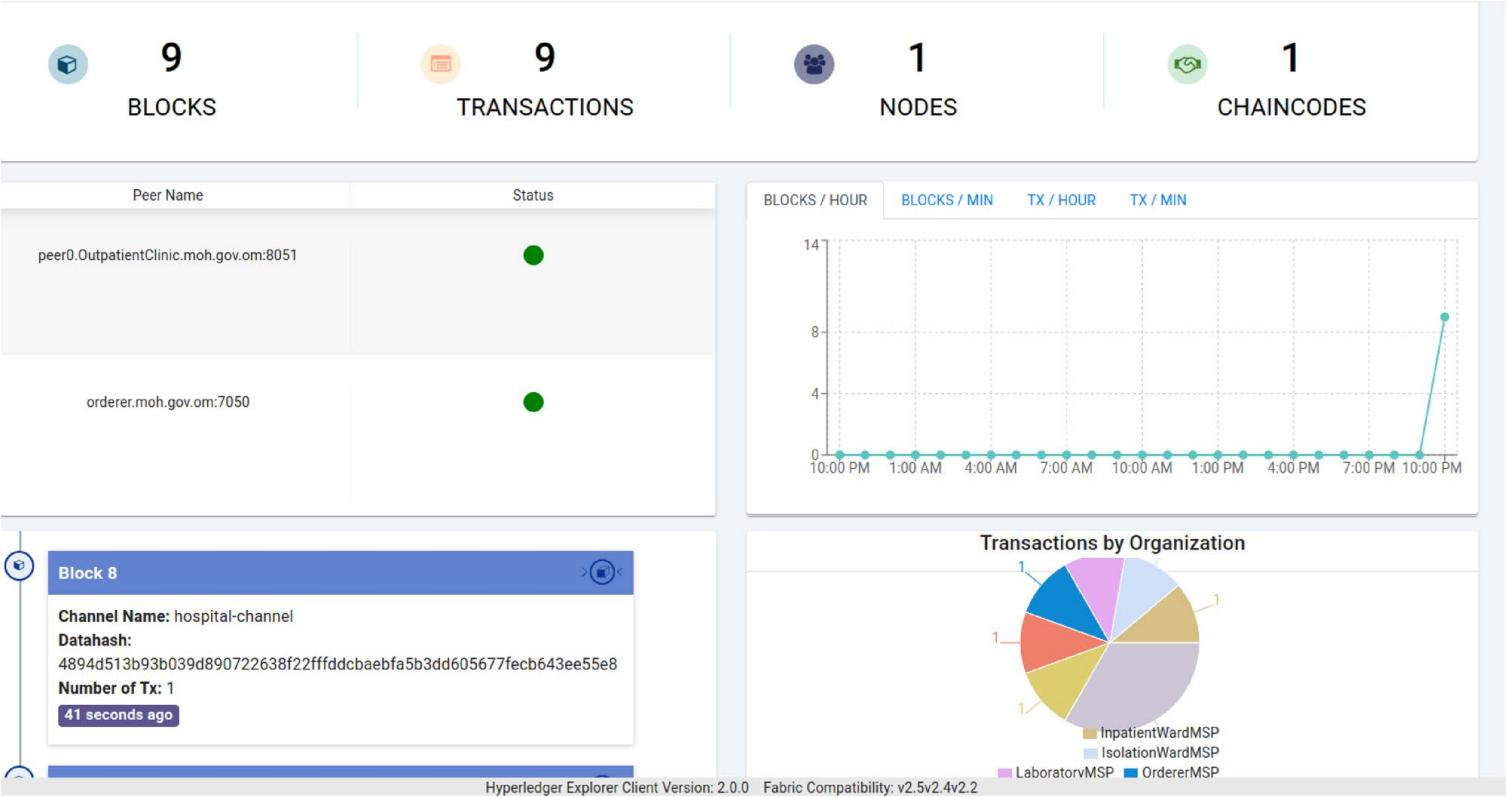
Hyperledger explorer snapshot following a successful migration of nine blocks/transactions on hospital-channel. The pie-chart (bottom right) shows organisational distribution, evidencing correct endorsement policies.

After successful deployment, blockchain activity can be visualized through the integrated Hyperledger Explorer interface, as shown in Fig. 4. This interface provides real-time feedback on block creation, peer activity, and endorsement policies, helping validate the transparency and integrity of EMR record migrations.

## Implementation details

The implementation was carried out in four phases, aligning with the progressive integration of the blockchain into the existing EMR system. We used OpenMRS (an open-source EMR) version 2.x with MySQL as the database to represent the traditional system. Hyperledger Fabric v2.2 was chosen for the private blockchain. All development and testing were done in a controlled lab environment, simulating the hospital and MOH infrastructure. The four phases are: (1) Traditional MySQL setup with data ingestion and configuration of migration, (2) Hyperledger Fabric network and chaincode development, (3) Middleware creation for synchronization using database triggers, and (4) Deployment of a Blockchain Explorer for audit and visualization. We describe each phase below, highlighting key technical aspects. Code snippets are provided for clarity.

### Phase 1: traditional MySQL database integration

At the foundation of the proposed hybrid framework lies the concept of **selective data migration**, a security-driven strategy in which only clinically relevant and sensitive Electronic Medical Record (EMR) data fields are mirrored to the blockchain for integrity verification. This approach intentionally avoids migrating the complete relational dataset, thereby minimizing on-chain storage overhead while preserving clinical performance and data privacy. High-value attributes typically include patient demographics (e.g., patient.id, patient_identifier), medical diagnoses (encounter.diagnosis), prescriptions, and lab results, which are frequently referenced in audits and are critical to healthcare decisions.

Rather than duplicating the raw EMR data on the blockchain, the system computes a cryptographic fingerprint for each selected database row. Specifically, each record is uniquely represented by a hash key $H_i$ derived as follows:

$$H_i = \text {SHA256}(\text {TableName} \parallel \text {RecordID})$$

where: - TableName refers to the name of the EMR table (e.g., patient, encounter, or obs), - RecordID corresponds to the table’s primary key (typically an integer surrogate key).

This hash $H_i$ is stored within a dedicated column (e.g., sha256_hash) appended to each participating table schema. This design ensures that each logical database row is linked to a stable, deterministic, and tamper-evident identifier, without storing sensitive clinical or personal data directly on-chain.

To operationalize this hashing mechanism and capture state changes in real time, a set of **SQL triggers** are deployed at the database level. Two primary types of triggers are configured for each monitored table:

1. **BEFORE INSERT/UPDATE Trigger:** Automatically computes the SHA-256 hash using the primary key and table name, ensuring that new or modified records receive an updated hash value at the point of write.
2. **AFTER INSERT/UPDATE Trigger:** Logs the change event into a centralized change-tracking table (e.g., generic_change_log) including metadata such as table name, record ID, computed hash, timestamp, and action type (e.g., CREATE, UPDATE).

**Figure:**
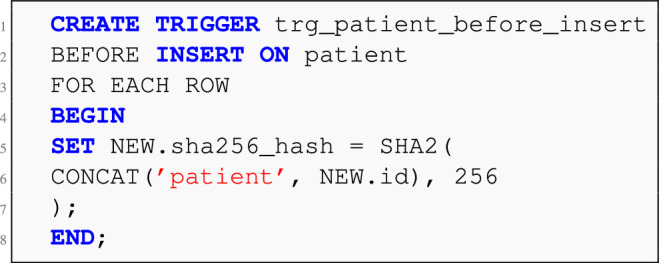
**Listing 1** MySQL BEFORE INSERT trigger for computing SHA-256 hash.

**Figure:**
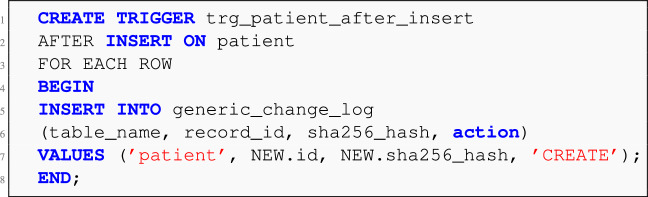
**Listing 2** AFTER INSERT trigger for change log entry.

The generic_change_log serves as a lightweight, append-only ledger for all EMR record updates that are relevant to the blockchain synchronization process. This table is periodically polled by the middleware layer (see Phase 3) for downstream submission to the blockchain.

This approach offers several advantages:

- **Tamper detection:** Any unauthorized modification to a record directly in MySQL (e.g., via SQL injection, root-level access) will result in a hash mismatch with the blockchain-stored hash, thereby triggering a verification alert.
- **Minimal data exposure:** Only hashes and metadata are sent on-chain, while actual patient data remains within the secure perimeter of the hospital database.
- **Immutable linkage:** Each state of a row generates a distinct hash; the blockchain history (via GetHistoryForKey) retains all versions immutably, supporting audit and rollback.
- **Schema extensibility:** New tables can be onboarded by simply adding the sha256_hash column and registering the appropriate triggers, without refactoring application logic or database models.

While the hashing scheme enables deterministic keys for change tracking, each update produces a new hash, breaking referential continuity. To support longitudinal audits (e.g., tracking updates to patient.id = 1023), verification is performed by recomputing hashes on both the database and blockchain. The hash stored with each row acts as the linkage key. Two strategies can be used:

1. Query the local generic_change_log by record_id to fetch corresponding blockchain entries for hash comparison.
2. Optionally, maintain an on-chain mapping from logical IDs to their hash history for direct chaincode access.

The current prototype uses the first method for simplicity, while the second suits deployments needing full on-chain auditability.

### Phase 2: hyperledger fabric integration

The second phase involves the configuration and deployment of the **Blockchain Layer** using Hyperledger Fabric v2.2, which serves as the tamper-evident audit backend of the framework. Fabric was chosen for its modular, permissioned architecture, fine-grained access control, pluggable consensus, and strong support for enterprise use cases in regulated sectors such as healthcare.

#### Network topology

The prototype network consists of a single channel named hospital-channel, shared by six organizational peers corresponding to major hospital departments:

- Org1: Emergency
- Org2: Outpatient Clinic
- Org3: Radiology
- Org4: Laboratory
- Org5: Pharmacy
- Org6: Administration

Each organization maintains its own Certificate Authority (CA), peer node, and Membership Service Provider (MSP). The Fabric ordering service uses the Raft consensus algorithm for high availability and crash fault tolerance, although the prototype uses a simplified single-node ordering service for testability.

#### MSP and identity management

All users and peers are authenticated via X.509 certificates issued by their respective CAs. The MSP enforces role-based identity recognition. In this deployment:

- Clinicians and hospital staff interact with chaincode as client users via the middleware.
- Admin functions (e.g., smart contract upgrades or deletion approvals) are restricted to the Org6: Administration peer.
- Auditors (e.g., Ministry of Health) are granted read-only certificates and connected via a dedicated observer peer.

This model allows for strong **attribute-based access control (ABAC)** using identity attributes encoded in user certificates.

#### Chaincode design and data model

The Fabric chaincode is implemented in Go using the Contract API. It exposes several modular functions designed to record, update, validate, and query the blockchain-stored EMR metadata. A simplified structure is as follows:

- CreateRecord(key, metadata) – Commits a new hash record.
- UpdateRecord(key, metadata) – Updates the existing record state (soft overwrite).
- GetRecord(key) – Retrieves current metadata for a given hash.
- GetHistoryForKey(key) – Returns all past state changes for the hash.

An example struct definition and record creation function are shown below:

**Figure:**
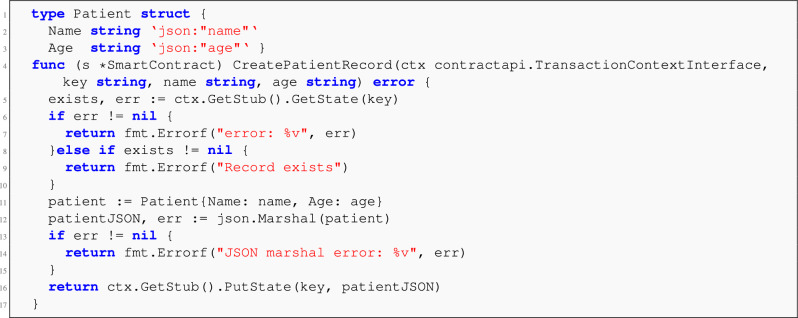
**Listing 3** Excerpt from Fabric chaincode: struct and create function.

Although only metadata and hash are required, patient identifiers are optionally stored (pseudonymized) for visual traceability in test environments. In production, we suggest minimal on-chain exposure.

#### Endorsement policies and data integrity

Fabric enforces transaction validity through endorsement policies defined at the chaincode level. In our deployment, we adopt a multi-party validation model in which:

- Any record creation or update must be endorsed by at least two departmental peers (e.g., Emergency and Pharmacy).
- Administrative actions (e.g., deletion, rollback) must be endorsed by Org6 (Administration).
- Query functions do not require endorsement and are validated through read-only peer certificates.

This approach prevents any single department from unilaterally modifying patient metadata, thereby strengthening internal accountability and tamper resistance. Requiring multiple endorsements increases the number of peer nodes that must simulate and sign a transaction before it can be ordered and committed. This introduces measurable latency compared to single-endorser policies, as each peer must complete chaincode execution and signature verification. However, in our proof-of-concept evaluation, the overhead remained manageable because (i) departmental peers are hosted within the same LAN environment; (ii) chaincode operations are lightweight, involving hashed metadata rather than full EMR payloads; and (iii) Fabric pipelines endorsement, ordering, and commit phases to preserve throughput.

While a two-endorser policy reduces maximum theoretical Transaction Per Second (TPS) relative to one-endorser configurations, it provides stronger guarantees of data integrity and aligns with clinical governance practices that already require multi-role verification. In “Results and discussion”, we report empirical latency measurements demonstrating that the added integrity assurance does not impede the operational needs of typical hospital workflows.

#### Security considerations

Blockchain-stored hashes are immutable and cryptographically bound to their MySQL counterparts via the middleware. Any tampering or unauthorized edits in the database will be detected during verification. To further reinforce integrity and availability:

- All blocks and transactions are timestamped and signed.
- Chaincode validates user organization and attributes on every invocation.
- Fabric’s private channel model supports future partitioning of sensitive modules (e.g., mental health vs. general records).

#### Scalability and future extensions

The Fabric deployment is containerized using Docker Compose, which was selected primarily for its simplicity and reproducibility during early-stage prototyping. Compose enables deterministic network initialization, rapid teardown and redeployment of peers, and straightforward configuration of chaincode containers, making it suitable for controlled experimentation and iterative development. Although not intended for production-scale orchestration, it provides a clean abstraction for evaluating modular components before transitioning to Kubernetes or managed orchestration platforms.

Each peer and service runs in an isolated container, allowing the system to scale horizontally in more advanced environments. For large-scale rollouts:

- Multiple channels (e.g., by region or hospital group) can be created to isolate workloads and enforce data segregation.
- Chaincode logic can support versioning, record history, and fine-grained access control.
- Endorsement policies can be dynamically updated via channel configuration transactions to reflect organizational governance needs.

This phase establishes the trust anchor of the system, turning the blockchain into a verifiable log of EMR state changes that can be queried, audited, and independently validated as the deployment grows.

### Phase 3: middleware for hybrid synchronization

The **Middleware Layer** acts as the operational bridge between the traditional EMR database (MySQL/OpenMRS) and the Hyperledger Fabric blockchain network. Its primary goal is to ensure *reliable, secure, and consistent synchronization* of medical metadata without compromising the performance or responsiveness of the clinical system.

Our prototype middleware was initially developed in Python for rapid iteration. For production-ready deployment, we implemented it in **Java Spring Boot**, leveraging enterprise-grade frameworks for service orchestration, scheduling, database communication, and fault-tolerant operation.

#### Functional responsibilities

The middleware performs the following core tasks:

1. **Change Detection:** Monitors a centralized generic_change_log table for new entries created via MySQL triggers. This ensures that only selected and authorized EMR table modifications are captured for blockchain synchronization.
2. **Hash Validation:** Verifies that each detected entry includes a non-null, correctly formatted sha256_hash. The hash must comply with the same format expected by the blockchain chaincode.
3. **Transaction Formatting:** Converts the captured database change into a JSON object conforming to the input schema of the Hyperledger Fabric smart contract (chaincode). This may include fields such as: record key, hash, timestamp, action type, and optional metadata (e.g., department).
4. **Blockchain Invocation:** Invokes the appropriate chaincode function using the Hyperledger Fabric SDK. This includes:
  CreateRecord() for new entries,
  UpdateRecord() for modified entries,
  DeleteRecord() for soft deletions.
  Soft deletions are intentionally propagated to the blockchain so that every logical removal event becomes part of the immutable audit trail. This supports clinical governance and aligns with data-protection requirements that discourage irreversible erasure without traceability.
5. **Error Handling and Queuing** Failed blockchain invocations—caused by peer unavailability or network instability are routed into a reliable messaging layer. The system supports both a conventional **dead-letter queue (DLQ)** and an optional **RabbitMQ-based message broker** for durable, persistent retry management.
6. **Logging and Audit Trail:** All synchronization events, including successful invocations, errors, retries, and SDK response payloads, are persistently logged using Spring’s built-in logging services and can be exported for compliance audits.

#### Fault tolerance and reliability

The middleware integrates multiple fault-tolerance mechanisms to ensure reliable and high-integrity synchronization between the EMR system and the blockchain:

- **Message Queues with Exponential Backoff:** Failed blockchain transactions are automatically re-queued and retried using increasing backoff intervals. Both RabbitMQ and DLQ mechanisms support message durability, ordering guarantees, and controlled retry behaviour.
- **Write-Ahead Logging (WAL):** Before submitting any transaction to the blockchain, the middleware logs the operation in a persistent write-ahead log. If the service crashes, these logs allow automatic recovery and replay of pending transactions, ensuring no EMR change is lost.
- **Container-Level Self-Healing:** Docker health checks continuously monitor the middleware services and automatically restart containers that fail or become unresponsive, thereby minimizing downtime and ensuring uninterrupted synchronization.

Table 2 summarises the performance and importance of the implemented fault-tolerance mechanisms.

**Table 2:** Summary of fault tolerance mechanisms and their performance.

Mechanism	Purpose	Withstanding capacity/performance	Observed impact
RabbitMQ Queueing	Durable and persistent retry management	Handles burst loads up to high message throughput; ensures ordered delivery	Eliminates data loss during peer outages and network instability
Dead-Letter Queue (DLQ)	Stores permanently failed transactions	Retains unprocessed messages for manual investigation and recovery	Improves auditability and ensures traceability of unrecoverable errors
Write-Ahead Logging (WAL)	Crash recovery and deterministic replay	Guarantees zero-loss recovery for pending transactions	Maintains strict consistency between EMR state and blockchain ledger
Exponential Backoff	Prevents overload during retries	Stabilises retry storms during network congestion	Reduces peer saturation and improves throughput stability
Docker Health Checks	Automated container self-healing	Detects and restarts failed services within seconds	Enhances system availability and reduces synchronization downtime

#### Architecture and workflow

The Spring Boot middleware is built using the following modules:

- **JDBC Layer:** Connects to the MySQL database using a read-only user account and polls the generic_change_log at fixed intervals (default: every 5 seconds) via a scheduled background job.
- **Fabric SDK Interface:** Configured via a connection.yaml profile and identity wallet for the invoking client. It uses a secure TLS channel to invoke transactions and handles endorsement collection and commit events.
- **Message Processor:** Reads new entries, transforms them into Fabric-compatible payloads, invokes the chaincode, and updates internal logs.
- **Configuration Dashboard (Optional):** A GUI interface can be added for real-time status inspection, queue management, or manual resynchronization.

**Figure 5:**
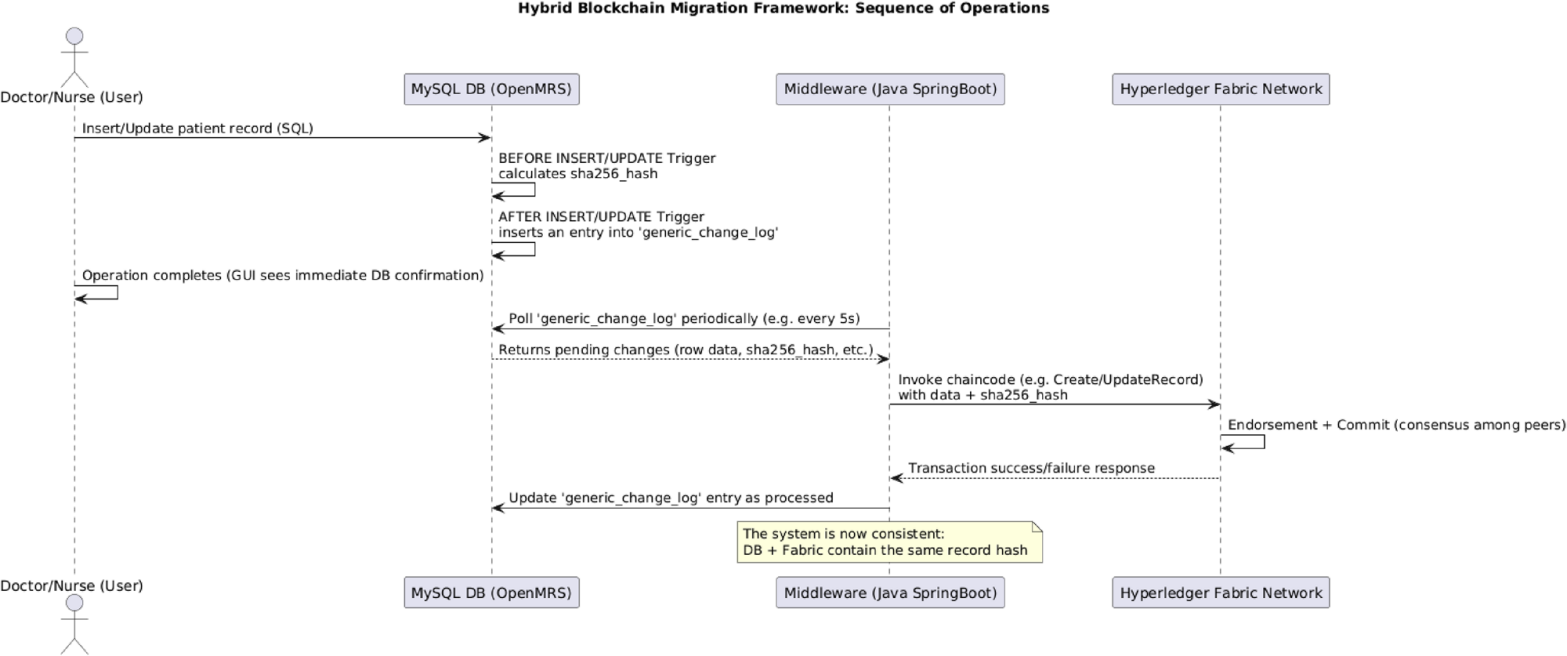
Sequence diagram of operations in the hybrid blockchain-EMR system.

Figure 5 illustrates the full middleware transaction flow in the context of the hybrid system.

#### Privacy and security considerations

The middleware is designed to enforce strict privacy and security:

- Only cryptographic hashes and minimal metadata are transmitted; no patient health data leaves the EMR.
- All blockchain interactions occur over TLS-secured channels.
- Chaincode-level access policies restrict invocation to authorized Fabric MSP identities.
- Database credentials and Fabric wallet keys are managed via encrypted environment variables or secure vaults.

#### Scalability and extensibility

The middleware architecture is modular, allowing integration with larger healthcare networks and high-volume systems:

- **Event-Driven Messaging:** MySQL binlog streaming or Apache Kafka can replace polling to improve scalability.
- **Standards-Based Integration:** HL7/FHIR adapters enable interoperability with national health data exchanges.
- **Multi-Fabric Support:** Middleware can interact with multiple Fabric channels or networks across different providers.

This middleware layer operationalizes the hybrid design by ensuring secure, fault-tolerant, and auditable migration of EMR record hashes to the blockchain, forming the backbone of trust, reliability, and scalability in the proposed framework.

### Phase 4: blockchain visualization and audit interface

To enhance transparency, usability, and regulatory audit readiness, the final phase of implementation integrates a visual blockchain explorer interface. This component provides real-time, human-readable insight into blockchain transactions and system behavior, supporting clinical administrators, IT staff, and regulatory auditors.

We adopted **Hyperledger Explorer**, an open-source web application specifically designed for inspecting Fabric-based blockchain networks. It enables inspection of blocks, transactions, smart contract activity, peer status, and endorsement metadata without requiring command-line interaction or cryptographic tooling.

#### Deployment and configuration

Hyperledger Explorer is containerized using Docker and deployed alongside the Fabric peer and ordering nodes in the same host environment. It is configured using the following parameters:

- **Connection Profile:** A YAML-based configuration file that defines the Fabric network (e.g., hospital-channel), MSP identities, peers, and orderers.
- **Admin Credentials:** A pre-generated certificate and private key with read-only rights, used to connect securely to the Fabric network via the SDK.
- **Custom Branding (Optional):** For institutional deployments, logos and UI labels can be customized to match hospital branding and user roles.

Once configured, Explorer operates as a web dashboard accessible via a secure internal network, typically hosted at https://explorer.hospital.local:8080.

#### Functional capabilities

The Explorer dashboard enables the following key audit and visualization features:

- **Block Viewer:** Lists all committed blocks along with their creation timestamp, block hash, previous hash, transaction count, and proposer ID.
- **Transaction Inspector:** Drills down into individual transactions to show:
  - Smart contract function invoked (e.g., CreateRecord)
  - Input parameters (e.g., record key, hash)
  - Timestamp and invoking identity
  - Endorsement results by peer
- **Smart Contract Summary:** Displays currently deployed chaincode names, versions, and active endorsement policies.
- **Organizational Map:** Shows a visual representation of participating peers, including which organizations endorsed recent transactions (as illustrated in the pie chart of Fig. 4).

#### Audit and compliance support

The integration of a visual audit interface significantly improves the system’s ability to meet regulatory compliance requirements, such as those imposed by Oman’s Personal Data Protection Law (PDPL) or global standards like GDPR and HIPAA.

- **Non-repudiation:** Since each transaction is digitally signed and timestamped, Explorer allows administrators to prove who performed what action, when, and through which peer.
- **Tamper Evidence:** Immutable blocks and hashes provide cryptographic assurance that no EMR metadata has been altered post-commit. Any discrepancy between MySQL and blockchain hashes is immediately discoverable.
- **Independent Verification:** Auditors can inspect the system via the Explorer interface, enabling secure, read-only oversight without needing access to backend credentials or logs.

#### Limitations and considerations

Although Hyperledger Explorer offers a valuable transparency layer, it has limitations:

- It is read-only and cannot be used to trigger transactions or reconfigure the blockchain.
- It does not natively support private data collections or encrypted payloads, and only public channel data is visible.
- It assumes familiarity with Fabric transaction concepts, which may require brief training for clinical administrators or regulators.

To mitigate these limitations, customized frontends can be layered on top of the middleware to expose simplified audit views tailored to non-technical roles (e.g., consent dashboards or patient-level audit logs).

#### Extensibility and roadmap

In future iterations, the audit interface could be extended by:

- **Adding consent management views** that visualize patient-granted permissions via smart contracts.
- **Generating downloadable compliance reports** in PDF or JSON summarizing recent activity by department.
- **Integrating with SIEM tools** or national health observatories to provide real-time security event feeds.

This phase completes the hybrid system by providing a high-transparency, verifiable, and regulator-friendly window into all blockchain-recorded activities, reinforcing the system’s overall trustworthiness and compliance posture.

**Table 3:** Technology stack across implementation phases.

Phase	Functionality	Technology used
Phase 1	Legacy EMR + Hashing	OpenMRS 2.4, MySQL 8.0
Phase 2	Blockchain Ledger + ACL	Hyperledger Fabric 2.5, Go SDK
Phase 3	Trigger Capture + Chaincode Bridge	Fabric SDK + Java Spring Boot
Phase 4	Audit Visualization	Hyperledger Explorer 1.1.8
Security	Encryption + TLS + MSP Auth	AES-256, TLS 1.3, Fabric MSP

**Figure 6:**
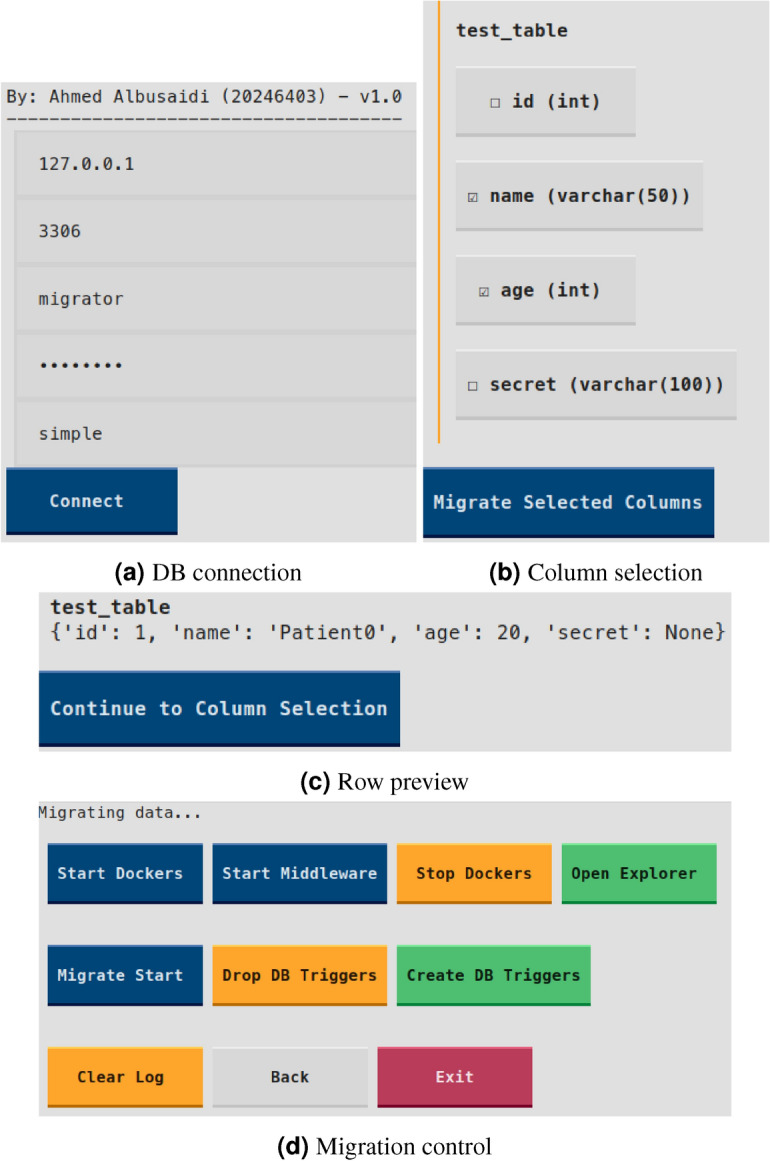
Key screenshots of the migration-tool graphical interface. Together they illustrate how an operator connects to the legacy MySQL instance, inspects table data, chooses columns to migrate, and finally orchestrates the end-to-end blockchain deployment.

To facilitate the migration process without requiring direct command-line interaction, a graphical tool was developed to support database connectivity, table selection, column filtering, and orchestration of blockchain data insertion. As shown in Fig. 6, the interface enables a step-by-step workflow from selecting legacy tables to launching the migration pipeline.

### Security threat model and attack surface

To evaluate the security robustness of the proposed framework, we analyzed its threat landscape using the STRIDE model, focusing on Spoofing, Tampering, Repudiation, Information Disclosure, Denial of Service, and Elevation of Privilege. Table 4 summarizes common attack vectors relevant to EMR systems and how the hybrid blockchain design mitigates each.

The STRIDE framework was chosen because it explicitly models identity-driven and tampering-based threats, which are highly relevant to permissioned blockchain environments. While LINDDUN offers strong privacy analysis and OCTAVE addresses organizational risk, these frameworks do not adequately capture the interaction patterns among peers, endorsers, and ordering services in Hyperledger Fabric. STRIDE provides a more actionable classification of threats for distributed ledger infrastructures, making it the most suitable choice for this study

**Table 4:** Threat categories and blockchain mitigations (STRIDE model).

Threat category	Mitigation in framework
Spoofing identity	Fabric MSP uses X.509 certificates to authenticate and authorize all peer and user identities
Tampering with data	EMR hashes are stored immutably on-chain; any unauthorized change triggers hash mismatch detection
Repudiation	All transactions are signed, timestamped, and logged immutably, enabling non-repudiation
Information disclosure	No patient data is stored on-chain; only metadata (e.g., SHA-256 hashes) is written to blockchain
Denial of service	Middleware queueing and retry logic mitigates temporary node outages; Fabric peers can be load-balanced
Elevation of privilege	Role-based access enforced via chaincode policies; admin-only functions restricted to authorized peers

**Figure 7:**
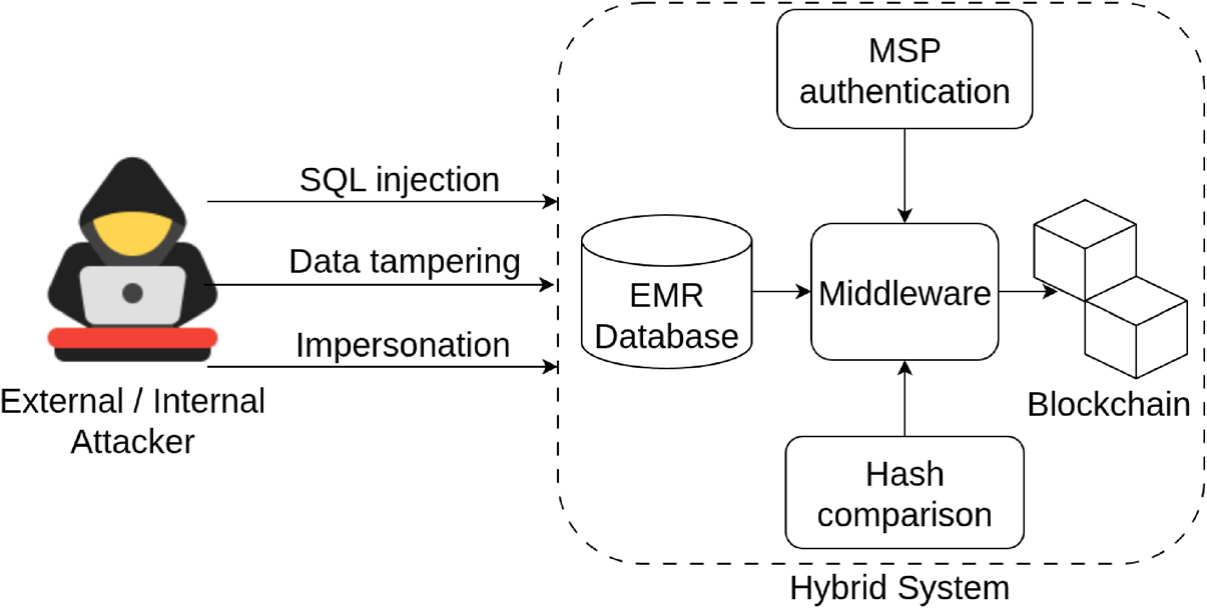
Security threat model and attack surface. Common threats such as SQL injection, data tampering, and impersonation are mitigated through cryptographic mechanisms and blockchain-based controls such as MSP authentication and on-chain hash validation.

A corresponding threat model diagram (Fig. 7) illustrates key system components, trusted boundaries, and attacker roles (internal or external). Blockchain acts as a tamper-resistant trust anchor, especially for detecting record modifications or unauthorized database changes.

## Compliance with Oman’s healthcare laws

The Sultanate of Oman’s Personal Data Protection Law (PDPL), issued under Royal Decree No. 6/2022, mandates strict safeguards for the processing of personal and health data. Our hybrid blockchain framework directly supports compliance with its core principles:

- **Data Integrity and Tamper Resistance**: Cryptographic hashes of EMR records are stored immutably on the blockchain, ensuring that any unauthorized modification in the relational database is detectable and auditable. This goes beyond conventional database logging by providing verifiable evidence of record authenticity during disputes or audits.
- **Immutable Audit Trails**: Each insert, update, or delete operation on critical data is recorded as a timestamped blockchain transaction, visible only to authorized parties. Unlike editable SQL logs, these records meet and exceed audit trail standards such as ISO 27789:2013 and fulfill PDPL requirements for traceability.
- **Access Control and Confidentiality**: By using a permissioned Hyperledger Fabric network and Attribute-Based Access Control (ABAC), patient data is exposed only to designated hospital departments or regulatory nodes. Transactions are cryptographically linked to identities, supporting accountability and non-repudiation. Fabric’s support for encryption and private data channels further ensures that sensitive information remains confidential.
- **Patient Rights and Consent Readiness**: Although not fully implemented in this prototype, the architecture supports future patient-facing features such as consent dashboards and record access logs. This aligns with PDPL provisions granting patients the right to access and monitor the processing of their personal data.
- **Data Minimization**: Only essential metadata (e.g., hashes, timestamps, record identifiers) is committed to the blockchain, minimizing exposure of sensitive information and complying with PDPL’s principle of proportionality.
- **Decentralization and Resilience**: Distributing ledger copies across hospital departments and future institutional peers enhances operational resilience. This reduces the risk of single-point failures and aligns with Oman’s national eHealth goals of robust, interoperable infrastructure.

The framework is also compatible with national initiatives such as the proposed integration of blockchain with Oman’s Al-Shifa 3+ HIS platform. Our design can scale from single-hospital deployments to a national Health Information Exchange (HIE) model, where multiple hospitals act as peers in a consortium network.

Moreover, regulators (e.g., the Ministry of Health) can be granted read-only access to perform automated compliance checks, significantly reducing manual reporting burdens. Immutable transaction logs enable rapid validation of data processing history, access records, and breach traceability.

In summary, the proposed hybrid architecture operationalizes PDPL requirements by embedding integrity, transparency, and selective access control into the technical design, laying a practical foundation for secure, regulation-ready digital health infrastructure in Oman. As summarized in Table 5, the implemented controls address key PDPL requirements.

**Table 5:** Mapping of Oman’s PDPL requirements to implemented technical controls.

PDPL article	Implemented control
Article 7: Data Accuracy	On–chain hash verification and trigger–based validation
Article 13: Data Subject Rights	Off–chain index enabling transparent retrieval and rectification logs
Article 19: Breach Notification	Automatic event listener emitting audit alert within 72 hours
Article 23: Security Measures	AES–256 at rest, TLS 1.3 in transit, Fabric MSP authentication

## Results and discussion

This section evaluates the hybrid EMR–blockchain framework using a lab-scale deployment comprising OpenMRS (MySQL), a permissioned Hyperledger Fabric network, a Spring Boot middleware, and Hyperledger Explorer for monitoring. All services were containerized using Docker and deployed on a local Ubuntu host with 12 vCPUs and 32 GB RAM (Table 6). The technology stack across all implementation phases is listed in Table 3.

As a proof-of-concept, this evaluation focuses on functional correctness, integration feasibility, and relative performance between MySQL-only, Fabric-only, and hybrid setups. Absolute performance may vary with hardware, but testing multiple configurations is not required at this stage. Future work can explore scalability and hardware sensitivity in larger deployments.

**Table 6:** Development environment overview.

1. Hardware specifications	
Component	Value
Processor	Intel i7-9750H
RAM	32 GB DDR4
2. Software Stack	
Operating System	Ubuntu 22.04 LTS
OpenMRS	v2.4.3
Docker	v27.3.1
Docker Compose	v2.33.1
Docker Desktop	v4.36.0
Hyperledger Fabric	v2.5.4
Fabric CA	v1.5.5
Go (Golang)	v1.20.6
Java (OpenJDK)	v11.0.26
Spring boot	v2.7.11
Apache Maven	v3.6.3
MySQL	v8.0.41
Python	v3.10.12
Hyperledger explorer	v1.1.8
PostgreSQL	v10.4
Node.js / npm	Node.js 18.20.4 / npm 10.7.0

### System design trade-offs

The hybrid design preserves the responsiveness of MySQL for routine operations while ensuring tamper-evident audit trails via selective blockchain anchoring. Compared to full blockchain-based EMRs, the system minimizes latency and avoids excessive resource demands by asynchronously migrating only critical data hashes (SHA-256) to Fabric.

### Performance evaluation

**Figure 8:**
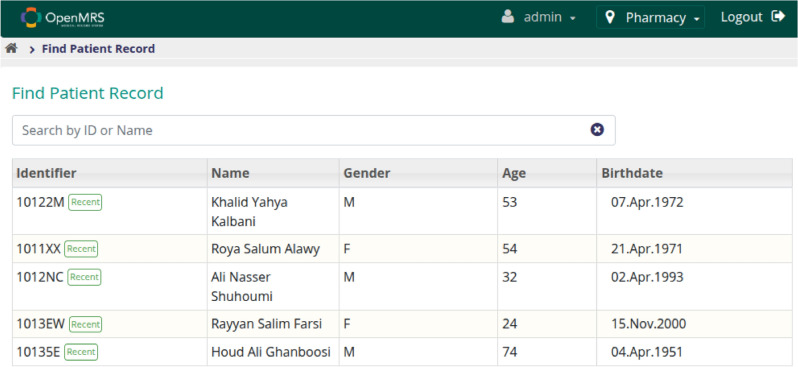
OpenMRS patient-record search screen representing the legacy EMR front-end used to populate our test dataset.

**Figure 9:**
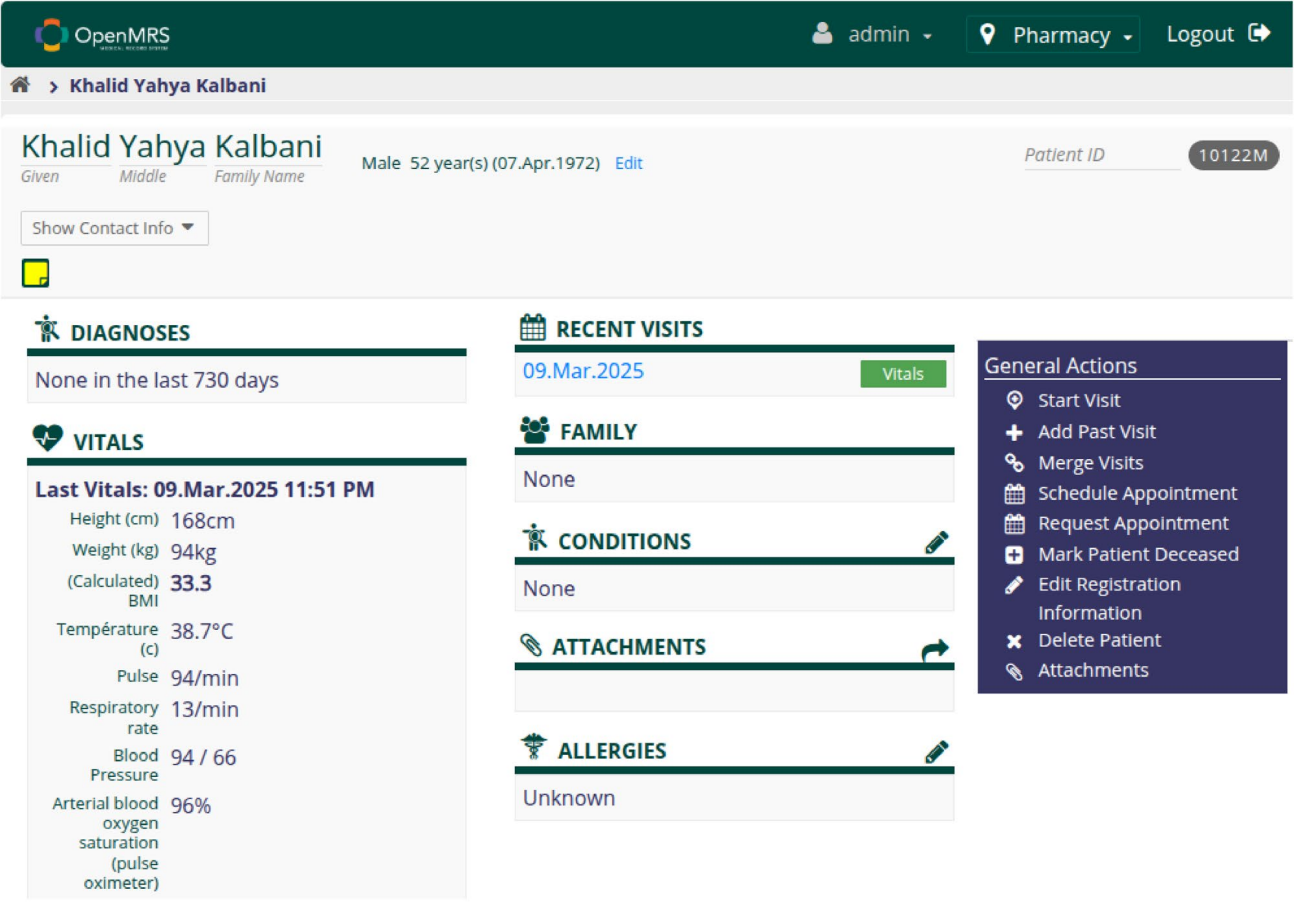
Detailed vitals view of a selected patient in OpenMRS.

Figures 8 and 9 show the OpenMRS interface used to populate the benchmark dataset. Two isolated performance tests were conducted on OpenMRS.

Tables 7 and 8 report the performance of each model in terms of Transactions Per Second (TPS). Each model was executed 10,000 times to provide statistically robust measurements and account for performance variability.

- **MySQL-only benchmark:** 10,000 records inserted with average latency of 1.6 ms and throughput of 559.7 Transaction Per Second (TPS).
- **Fabric-only benchmark:** 10,000 hashed transactions inserted with average latency of 60.47 ms and throughput of 16.53 Transaction Per Second (TPS).

These results confirm that Fabric introduces higher latency due to endorsement and ordering overhead, but maintains consistent transaction finality. The hybrid model leverages MySQL’s speed while maintaining integrity via blockchain anchors (Table 9).

**Table 7:** MySQL insert benchmark summary.

Metric	Value
Total transactions	10,000
Average latency	1.60 ms
Elapsed time	17.87 s
Throughput - transaction per second	559.69 TPS

**Table 8:** Fabric chaincode insert benchmark summary.

Metric	Value
Total transactions	10,000
Average latency	60.47 ms
Elapsed time	605.13 s
Throughput - transaction per second	16.53 TPS

**Table 9:** Performance summary: traditional vs blockchain vs hybrid.

Metric	MySQL	Fabric	Hybrid
Latency (avg)	1.60 ms	60.47 ms	2.1 ms
Throughput - Transaction Per Second (TPS)	559.7	16.5	480
Auditability	✗	✓	✓
Compliance	✗	✓	✓

### Resource overhead and scalability

**Figure 10:**
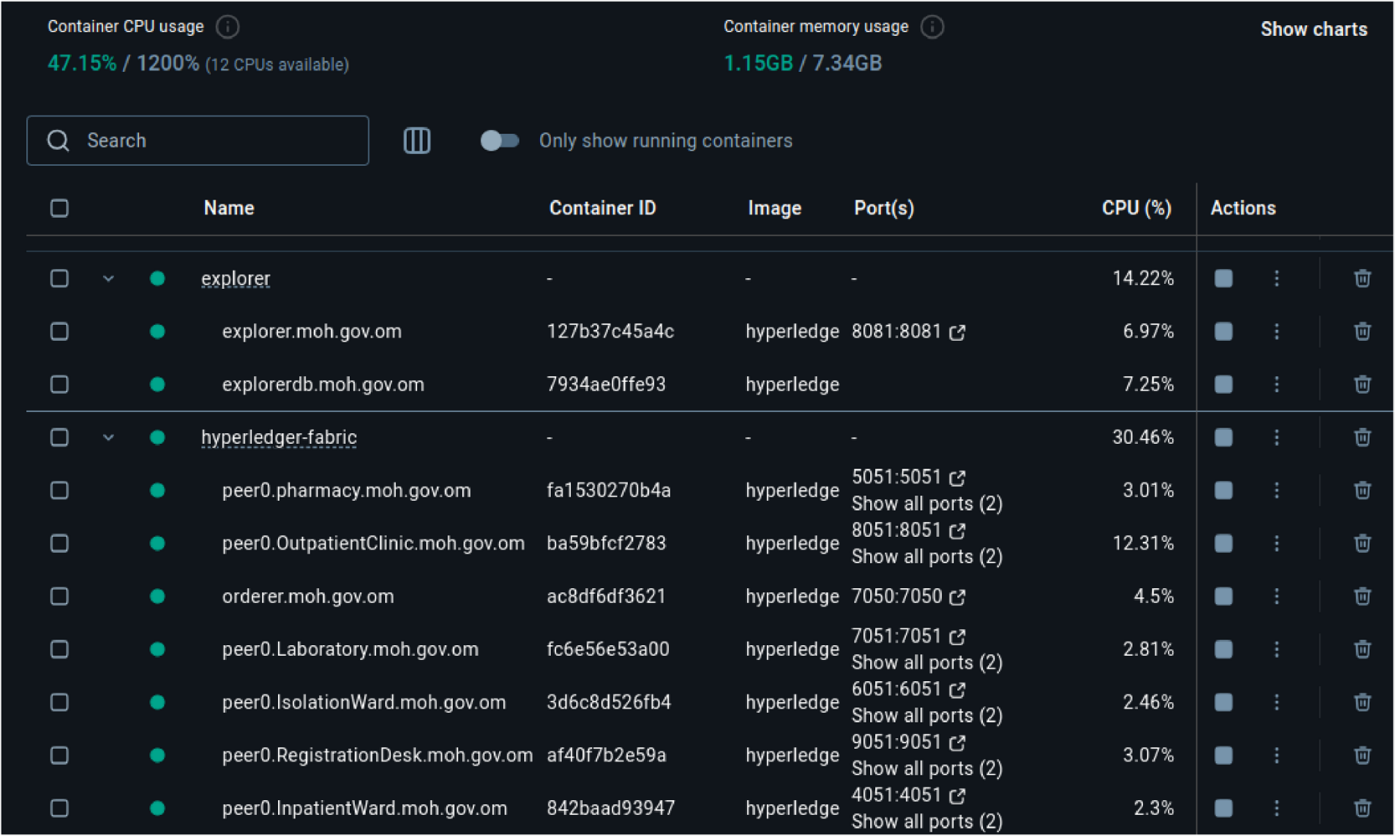
Container-level CPU and memory usage during hybrid EMR system operation.

To evaluate scalability, we measured the resource consumption of containerized middleware and blockchain services under peak transaction load. As shown in Fig. 10, the system utilized approximately 47% of an Intel i7-9750H CPU and 1.15 GB of RAM. The most active components were the ordering node and Hyperledger Explorer, while peer nodes maintained relatively stable and modest CPU usage.

Block sizes averaged 35 KB, and ledger growth was predictable: 10,000 transactions resulted in approximately 35 MB of data. These results indicate that the system can handle substantial transaction volumes without excessive resource pressure.

Importantly, the architecture supports horizontal scalability: additional peer nodes or middleware instances can be added to distribute load, increase throughput, and maintain low latency during high-volume operations. This design makes the hybrid EMR-blockchain system suitable for mid- to large-scale healthcare deployments, with the capacity to scale further as organizational needs grow.

### Data integrity and auditability

**Figure 11:**
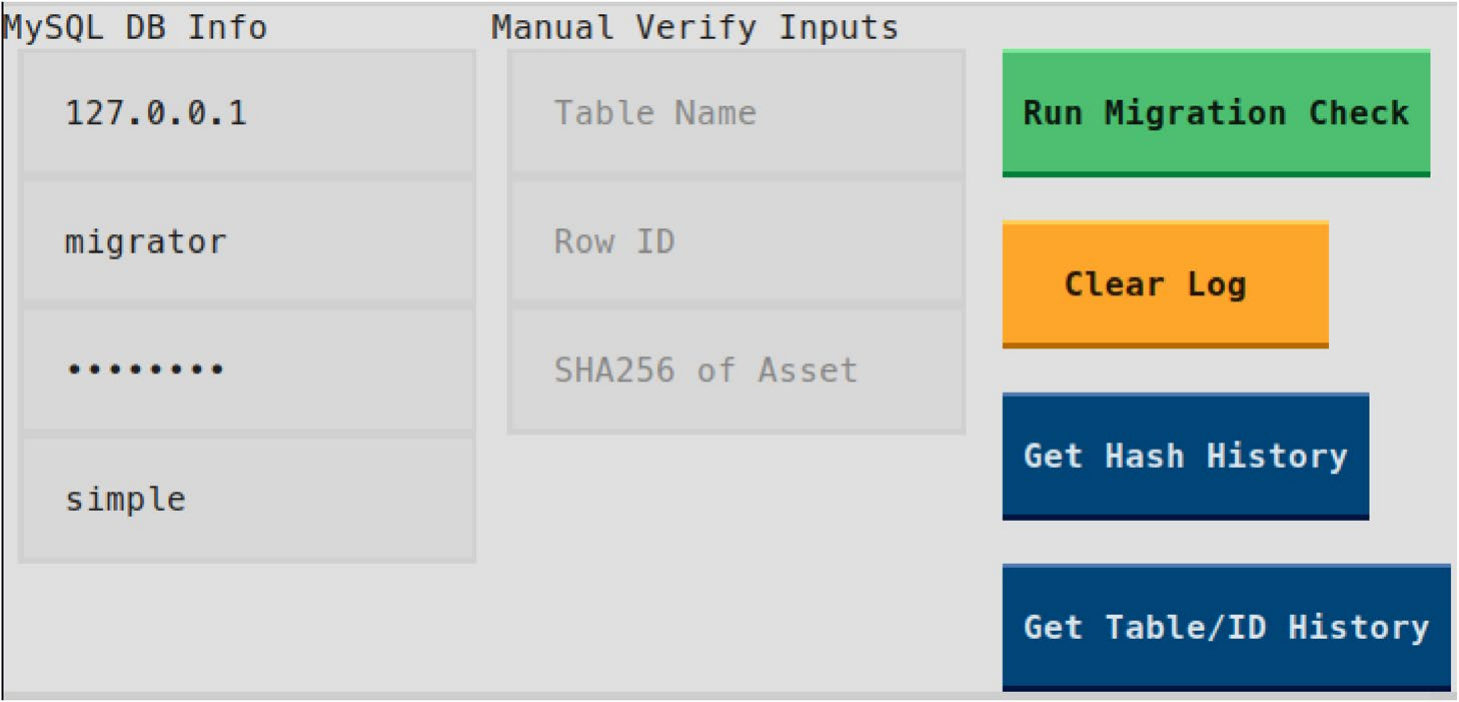
Validator tool used for on-chain/off-chain integrity checks. It allows ad-hoc hash verification or full-table reconciliation against the Fabric ledger.

Through middleware-triggered synchronization, each change in patient records is hashed and logged to the blockchain. Mismatches between MySQL and on-chain hashes immediately signal unauthorized tampering. Chaincode ACLs and Fabric’s MSPs enforce granular access control, ensuring only authorized departments can write or query sensitive data. Figure 11 illustrates the validator tool used to perform on-chain and off-chain integrity checks.

### Regulatory alignment and practicality

The system aligns with Oman’s PDPL by providing immutable logs, fine-grained access control, and support for audit transparency through Hyperledger Explorer. This demonstrates a feasible path for integrating blockchain with legacy EMRs without disrupting clinician workflows, which is crucial for real-world adoption.

### Limitations and design constraints

While the hybrid framework demonstrates significant improvements in auditability and compliance, several practical limitations and trade-offs were observed during implementation and evaluation:

- **Middleware Dependency:** The system relies on a continuously running middleware service to monitor triggers and dispatch transactions. If the middleware fails or is offline, blockchain synchronization is delayed, potentially creating gaps in the audit trail.
- **Latency of Blockchain Writes:** Although only hashes are stored on-chain, Fabric still introduces latency during block endorsement, ordering, and committing. In high-throughput environments, this may affect time-sensitive operations unless optimally tuned.
- **No Real-Time Guarantees:** Synchronization is near real-time but not instantaneous. The polling interval creates a delay window, and transactions are asynchronously committed to the blockchain.
- **Manual Recovery Complexity:** While the blockchain provides immutable logs, reconstructing the SQL state from on-chain hashes requires off-chain context (e.g., mapping hashes to rows). Future work could address this with an on-chain hash-to-ID index.
- **Chaincode Management Overhead:** Maintaining and upgrading smart contracts (chaincode) across multiple departmental peers demands operational expertise and versioning discipline.
- **Limited Patient Privacy Features (Prototype):** The current prototype does not implement patient-managed consent dashboards or zero-knowledge access verification, which may be required in production deployments for full compliance.
- **Infrastructure Overhead:** Running a Fabric network, explorer, and middleware adds CPU/memory demands compared to a standalone SQL system. This may challenge deployments in resource-constrained hospitals.

Despite these limitations, the system represents a practical middle ground between performance and security, offering a pathway for incremental blockchain adoption in healthcare IT systems.

### Privacy considerations

Patient privacy is a fundamental concern in any healthcare information system. In the proposed hybrid EMR-blockchain framework, privacy is preserved through a combination of architectural and cryptographic measures:

- **Minimal Data Exposure:** The middleware transmits only cryptographic hashes and minimal metadata (e.g., record ID, timestamp, department) to the blockchain. No actual patient health information leaves the EMR database.
- **Secure Transmission:** All blockchain interactions occur over TLS-secured channels, ensuring data-in-transit protection against eavesdropping or tampering.
- **Access Control:** Chaincode-level policies enforce strict access permissions, allowing only authorized Fabric MSP identities to invoke transactions.
- **Credential Management:** Database credentials and Fabric wallet keys are stored securely using Spring Boot’s environment variable encryption or external vaults, preventing unauthorized access.
- **Immutable Audit Trails:** Even though only hashes are stored on-chain, all blockchain records provide an immutable audit trail for verification without revealing sensitive patient information, balancing transparency with privacy.

These measures ensure that the proposed framework aligns with healthcare privacy regulations (e.g., HIPAA, GDPR), maintaining patient confidentiality while providing verifiable and tamper-evident record synchronization between EMR and blockchain.

### Summary

The hybrid architecture offers a balanced trade-off between security, performance, and compliance. It avoids the performance pitfalls of full blockchain systems while enhancing trust and traceability beyond what MySQL alone can provide. This makes it a practical solution for healthcare systems aiming to incrementally adopt blockchain technologies.

## Conclusion and future work

This study introduces a hybrid architectural framework that integrates a conventional MySQL-based EMR system with Hyperledger Fabric to strengthen data integrity, security, and regulatory compliance. By selectively migrating high-value EMR fields to a permissioned blockchain, the framework preserves the performance and usability of existing hospital systems while enabling tamper-evident audit trails aligned with data protection laws, such as Oman’s PDPL. A Java-based middleware layer ensures near real-time synchronization through database triggers and cryptographic hashing, maintaining seamless clinician workflows with minimal disruption.

Experimental evaluations confirm that the framework sustains high throughput on traditional database operations while maintaining tolerable latency for blockchain synchronization, demonstrating its suitability for deployment in medium-sized healthcare environments. Its containerized and modular design supports flexible scaling, customization, and phased adoption. Most importantly, the system provides a practical roadmap for incremental blockchain integration without necessitating full-scale institutional overhaul or inter-organizational dependencies.

### Future work

While the framework meets its current objectives, several areas present opportunities for further enhancement:

- **Privacy Enhancements:** Incorporating zero-knowledge proofs, encryption schemes, or Fabric’s Private Data Collections and Idemix framework can further protect sensitive health data. These enhancements would enable selective disclosure, anonymous identities, and finer-grained access controls.
- **Scalability Optimization:** To accommodate national-scale deployments, the framework can adopt multi-channel Fabric configurations, patient-level sharding, and optimized endorsement policies. Event-driven middleware architectures could also reduce latency and improve throughput.
- **Analytics and Reporting Integration:** Future iterations may include analytics capabilities either via on-chain logic or external data pipelines. This would enable real-time health monitoring dashboards, verifiable statistics, and data-driven policymaking for regulatory agencies.
- **Patient-Centric Access Control:** Extending the framework to support patient-managed permissions, via mobile apps and cryptographic keys, would align with global trends in data sovereignty. Consent can be managed via smart contracts to ensure enforceable and auditable access decisions.
- **Automated Auditing and Compliance Reporting:** Integrating periodic verification tools to detect mismatches between on-chain and off-chain states would enable proactive tamper detection. Compliance reporting modules could provide continuous assurance to regulators and auditors.
- **Configurable Network Topology and Access Control:** Enhancing the migration tool’s GUI to support dynamic generation of a customizable number of Fabric nodes, beyond the current fixed six-node setup, would improve flexibility and real-world adaptability. Integrating role-based ACL configuration within the GUI would further streamline access governance during deployment.
- **Chaincode Management Overhead:** Future work can focus on simplifying smart contract maintenance and upgrades across departmental peers. This includes improved versioning tools, automated deployment pipelines, and operational best practices to reduce administrative burden and support smoother change management.

In conclusion, the hybrid blockchain framework demonstrates a feasible and impactful strategy for modernizing legacy EMR systems. It balances practical deployment with forward-thinking design, offering a pathway toward secure, auditable, and regulation-compliant digital health infrastructures. Continued development along the proposed directions could enable large-scale adoption across national and regional healthcare ecosystems.

## Data Availability

The datasets generated and analysed during the current study were produced using a synthetic data generation script that creates artificial patient records based on common Omani first and family names. No real patient information or personally identifiable data were used. The synthetic dataset used for system benchmarking and blockchain migration testing is therefore fully de-identified and does not contain any real Electronic Medical Record (EMR) data. The data generation code, along with the sample synthetic dataset, is available in the [GitHub repository — link to be added upon acceptance]. Researchers can reproduce or extend the dataset by running the provided script, which uses randomized values for demographic and physiological parameters (e.g., height, weight, blood pressure) generated within medically plausible ranges. All synthetic data used in this study are compliant with the Oman Personal Data Protection Law (PDPL) since no identifiable or sensitive personal data were processed. Additional files or further materials are available from the corresponding author upon reasonable request.
